# Anticoagulant Therapy in Neonatal Acute Infectious Peritonitis Based on the TAT, PIC, t-PAIC, and sTM: A New Case Series

**DOI:** 10.1055/a-2770-6902

**Published:** 2025-12-31

**Authors:** Wenya Wang, Yue Gao, Yan Qiao, Yang Wu, Jiao Li, Li Zhang

**Affiliations:** 1 Department of Pediatrics, Sichuan University West China Second University Hospital, Chengdu, Sichuan, China; 2 Laboratory of Birth Defects and Related Diseases of Women and Children, Sichuan University, Ministry of Education, Chengdu, China; 3 Department of Pediatrics, Shanxi Coal Central Hospital, Taiyuan, Shanxi, China; 4 Department of Pediatric Surgery, Sichuan University West China Hospital, Chengdu, Sichuan, China

**Keywords:** LMW-heparin, hypercoagulability, infectious diseases, acute peritonitis, TAT, PIC, sTM, t-PAIC

## Abstract

Neonatal acute peritonitis is a leading cause of morbidity and mortality and poses challenges that demand prompt diagnosis and treatment, particularly in infants with disseminated intravascular coagulation. Here, we report a case series of four infants with acute peritonitis caused by necrotizing enterocolitis, gastrointestinal perforation, and meconium peritonitis. Laboratory tests for thrombin–antithrombin III complex (TAT), plasmin-α2-plasmin inhibitor complex (PIC), soluble thrombomodulin (sTM), and tissue plasminogen activator–inhibitor complex (t-PAIC) suggested the activation of the coagulation system followed by treatment with anticoagulant therapy in these infants. Overall, TAT, PIC, t-PAIC, and sTM may guide anticoagulant therapy, offering prospects for improving the outcomes in neonates with acute peritonitis.

## Introduction

Neonatal acute peritonitis is a major cause of neonatal acute abdomen and is a challenging problem that requires timely diagnosis and treatment. The reported morbidity rate of acute peritonitis in newborns is approximately 1.67% with causes including necrotizing enterocolitis (NEC), gastrointestinal perforation, and meconium peritonitis. 1 2 3 Although the mortality of neonatal acute peritonitis has gradually decreased from 99% in 1939 to 33% in 1983 with the improvement of medical technology, mortality remains high and varies by the conditions causing peritonitis, with NEC associated with 33% of cases and meconium peritonitis associated with 19.1%. 1 2 Among these, microcirculation disturbances, especially disseminated intravascular coagulation (DIC), are important causes of neonatal mortality in acute peritonitis. 2 Hayato et al 4 suggested that almost 30% of neonates with DIC have gastrointestinal perforations with NEC. Therefore, acute neonatal peritonitis is associated with a high risk of DIC development and high mortality. However, early standard detection and intervention for coagulation in newborns with acute peritonitis are lacking. Recognition of this special condition will enable early diagnosis and anticoagulation treatment, which could improve prognosis. Recently, the clinical efficacy of thrombin–antithrombin III complex (TAT), plasmin-α2-plasmin inhibitor complex (PIC), soluble thrombomodulin (sTM), and tissue plasminogen activator–inhibitor complex (t-PAIC) has been proven in sepsis and sepsis-induced coagulopathy; however, reports of these markers in the newborn are rare. 5 6 We present four cases of neonatal acute infectious peritonitis from different causes that were examined for the coagulation markers TAT, PIC, t-PAIC, and sTM. The diagnostic values and clinical outcomes were evaluated. A preprint version of this manuscript has been previously posted online. 7

## Case Reports

### Case 1

**Fig. 1:**
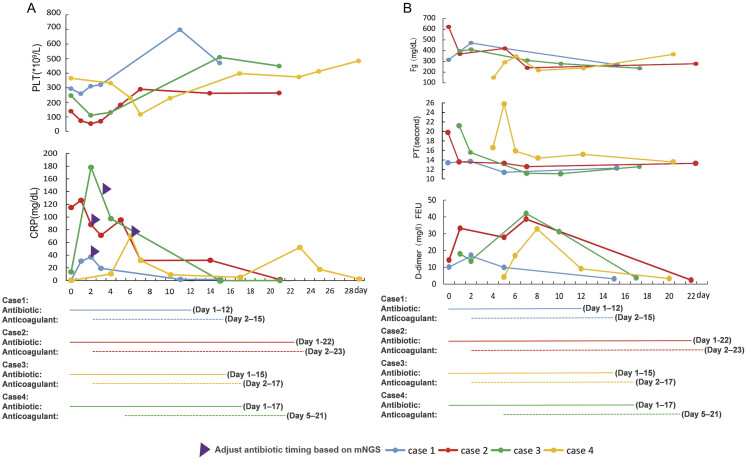
( **A** ) The levels of CRP and platelets PLT of four patients during hospitalization. The total antibiotic courses and adjustments of antibiotics based on the pathogen metagenomic next-generation sequencing (mNGS) results from ascites or blood in the four patients are shown. ( **B** ) The levels of D-dimer, PT, and Fg during the hospitalization of four patients. CRP, C-reactive protein; PLT, platelet count; PT, prothrombin time; Fg, fibrinogen.

A 3-day-old full-term infant with a history of severe perinatal asphyxia was transferred to our hospital because of “emesis, lethargy, and abdominal distension for 4 hours.” On the day of admission, he presented with bilious vomiting when 30 mL/kg of enteral feeding was achieved. He developed bloody stools, fever, and weakened bowel sounds. The diagnosis of stage IIIA NEC was confirmed using clinical signs, radiography, and ultrasonography. TAT levels markedly increased, whereas sTM levels mildly increased. Additional test results are shown in Fig. 1 . He received antibiotics for 12 days and anticoagulant therapy with enoxaparin for 14 days and was discharged without complications.

### Case 2

**Fig. 2:**
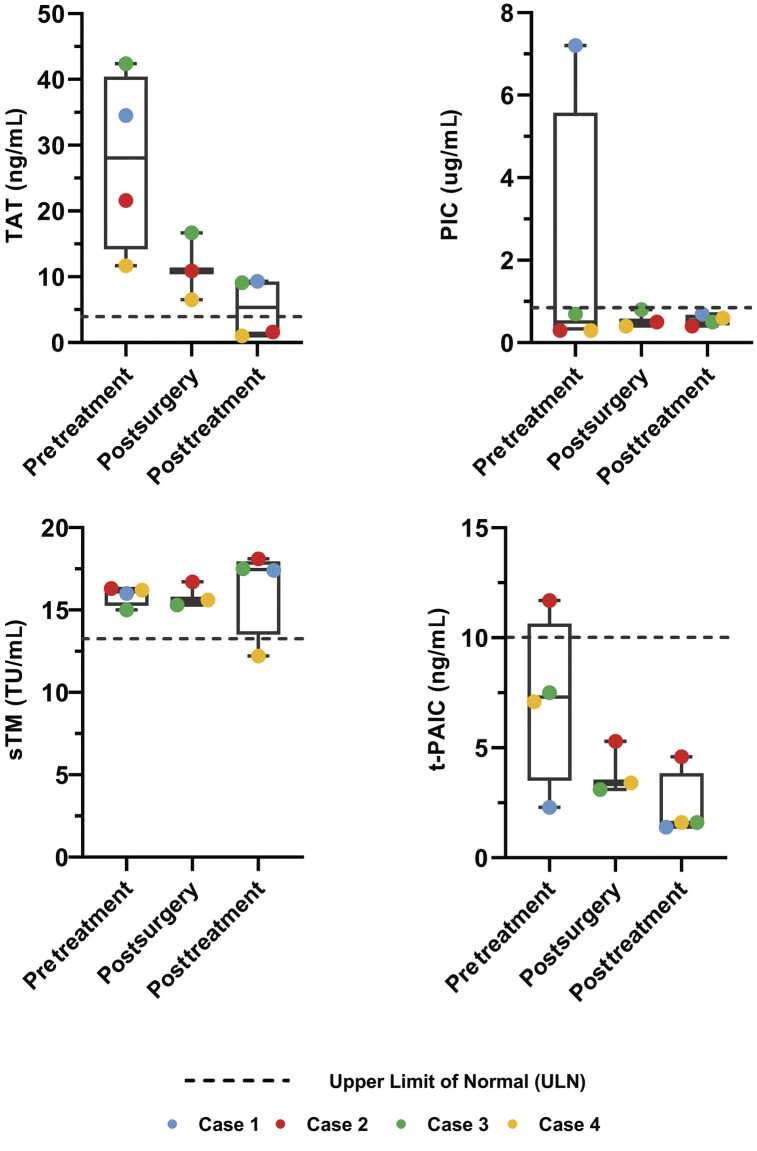
The levels of TAT, PIC, sTM, and t-PAIC during the hospitalization of four patients. “Pretreatment,” “postsurgery,” and “posttreatment” denote blood samples collected at distinct time points following disease onset: cases 1 (days 1 and 15; this patient did not undergo surgery), 2 (days 1, 4, and 24), 3 (days 2, 5, and 18), and 4 (days 4, 6, and 20). Dotted lines indicate the upper limits of normal for each biomarker: TAT < 4.0 ng/mL, PIC < 0.8 μg/mL, t-PAIC < 10.0 ng/mL, and sTM < 13.3 TU/mL. TAT, thrombin–antithrombin III complex; PIC, plasmin-α2-plasmin inhibitor complex; sTM, soluble thrombomodulin; t-PAIC, tissue plasminogen activator–inhibitor complex; ULN, upper limit of normal.

An 8-day-old preterm infant was hospitalized with fever, abdominal distension, and hematochezia. At 7 days of age, the patient achieved full enteral feeding with breast milk plus a cow milk-based formula and developed symptoms. The full blood counts, coagulation and fibrinolysis markers are listed in Figs. 1 and 2 . Ileocolic resection and ileostomy were performed, and the patient was diagnosed with stage IIIB NEC, and the levels of TAT and sTM also increased. Therefore, enoxaparin was administered as an anticoagulant for 23 days. Trophic feeding began on the fifth day after the operation, and oral feeding was established after 14 days.

### Case 3

A 3-day-old full-term infant with tracheal intubation was transferred to our hospital because of “emesis and abdominal distension for 3 days.” The patient started to vomit after bottle feeding 6 hours after birth, accompanied by progressive abdominal distension and failure to pass meconium. Physical examination revealed skin mottling, high abdominal distension, elevated skin temperature, slight abdominal wall discoloration, weak bowel sounds, and a capillary refill time of 4 to 5 seconds. The laboratory examination showed that the white blood cell counts decreased, CRP slightly increased, TAT significantly increased, and sTM mildly increased, indicating hypercoagulability. The patient was diagnosed with meconium peritonitis postoperatively. Anticoagulation therapy with enoxaparin was administered for 15 days. The length of hospital stay was 23 days.

### Case 4

The newborn, aged 24 minutes, was admitted to our department because of premature birth. On the fourth day after birth, the patient presented with vomiting, abdominal distension, tachypnea, and groaning with minimal feeding. Physical examination revealed abdominal enlargement, high abdominal distension, and the absence of bowel sounds. The patient was diagnosed with a congenital gastric wall defect postoperatively. On the first day after surgery, TAT increased, suggesting hypercoagulability, and enoxaparin was administered as an anticoagulant for 16 days. The mean time to full enteral feeding was 11 days. The length of hospital stay was 30 days.

All patients were discharged from the hospital and tolerated feeding with normal coagulation test results. The clinical characteristics of the four patients are presented in Table 1 . The levels of PLT and CRP are shown in Fig. 1A . The levels of traditional coagulation markers, including prothrombin time (PT), fibrinogen, and D-dimer are shown in Fig. 1B . Levels of TAT, PIC, sTM, and t-PAIC in the four patients are shown in Fig. 2 . In infants with acute infectious peritonitis who underwent surgery, detection time points are usually three times, one before the surgery, another 24 to 48 hours after surgery, and a third 10 to 14 days after surgery. For infants with NEC receiving medical treatment who did not undergo surgery, testing time is conducted on the day of onset and again at 10 to 14 days. If the re-examination still shows abnormal results, we will continue with follow-up examinations approximately every 5 to 7 days based on the clinical situation. The sampling time points have now been clearly stated in the legend of Fig. 2 , indicating the days of blood collection for each case. Laboratory tests for TAT, PIC, sTM, and t-PAIC were normal in four control cases, including one case of a high-type imperforate anus with ostomy surgery, two cases of intestinal stenosis surgery following NEC, and one case of food protein-induced allergic proctocolitis.

**Table 1:** Clinical characteristics of four cases

	Case 1	Case 2	Case 3	Case 4
Admission, month, and year	October 11, 2023	October 10, 2023	October 31, 2023	October 10, 2023
Age at admission (d)	3	8	3	0
GA (wk)	37 ^1/7^	36	39 ^4/7^	32 ^4/7^
BW (g)	3,700	2,125	4,000	1,840
Sex	girl	Girl	boy	boy
Apgar 1–5 min	2–9	9–10	10–10	9–10
Signs and symptoms	Emesis, lethargy, abdominal distension, hematochezia, and fever	Fever, abdominal distension, and hematochezia	Emesis, abdominal distension, failure to pass meconium, and poor perfusion	Emesis, abdominal distension, tachypnea, and groaning
Operative findings	–	The full length of the small intestine was about 80 cm; the ileum perforation was 5 cm from the ileocecum	Some of the intestinal tubes adhered in a mass, and pus was attached to the surface of the intestinal tubes; perforation of the ascending colon of 0.5 cm in diameter was seen 2 cm from the ileocecal portion	A huge 8 cm muscular layer defect of the gastric wall at the greater gastric curvature
Diagnosis	NEC IIIA	NEC IIIB	Meconium peritonitis	Gastric wall defect
X-ray	Portal venous gas	Pneumoperitoneum	Accumulation of free gas in the abdominal cavity	Pneumoperitoneum
Pathogen (mNGS of ascites)	Clostridium butyricum	Fecal coliform, Enterococcus faecalis, Escherichia coli	Klebsiella pneumoniae, Enterococcus faecalis, Acinetobacter baumanii	Streptococcus, Enterobacter cloacae
Blood culture	Negative	Negative	Enterococcus faecium	Negative
Cerebrospinal fluid	–	White blood cell count: 27 × 10⁶/L; glucose: 1.97 mmol/L; protein: 1,088.4 mg/L; nucleic acid testing: negative	Normal	–
Antibiotics after mNGS	Cefmetazole and ampicillin	Meropenem (for 15 d) + vancomycin (for 23 d)	Meropenem (for 6 d) + vancomycin (for 14 d)	Penicillin (for 11 d) + meropenem (for 13 d)
Duration of mechanical ventilation (d/h)	–	MV (for 5 d)	MV (for 6 d) + HHFNC (for 2 d)	HHFNC (for 2 d) + IMV (for 8 d) + nCPAP (for 1 d)
Vasoactive drugs	–	Dopamine (for 2 d)	Dopamine (for 1 d) + epinephrine (for 6 d)	Dopamine (for 1 d) + dobutamine (for 10 d) + norepinephrine (for 5 d)
Length of stay (d)	20	26	23	30

Abbreviations: BW, birth weight; GA, gestational age; HHFNC, heat-humidified high-flow nasal catheter; IMV, invasive mechanical ventilation; mNGS, metagenomic next-generation sequencing; MV, mechanical ventilation; nCPAP, nasal continuous positive airway pressure; NEC, necrotizing enterocolitis; WBC, white blood cell.

## Discussion

Here, we reported four cases of acute diffuse peritonitis in newborns receiving anticoagulation treatment. The pathophysiology of DIC in newborns begins with endothelial damage caused by various primary illnesses. Many studies suggested that an additional problem in neonatal peritonitis that should be noted is DIC, which could further aggravate cell and tissue injury or even death. 2 Since nonovert DIC is the stage prior to overt DIC, early recognition and treatment of nonovert DIC might improve prognosis. An article on the pediatric sepsis-induced coagulation score suggests that, with markedly elevated CRP levels in all four of our diffuse peritonitis cases, we also considered infection-induced coagulopathy. 8 Previous studies have revealed that TAT can be used as a biomarker for nonovert DIC for early diagnosis and anticoagulation treatment; otherwise, patients might lose the therapeutic window and develop overt DIC with severe thrombocytopenia and abnormal activated partial thromboplastin time, PT, and fibrinogen. 6 However, studies on the sensitivity of nonovert DIC diagnostic criteria for neonatal acute diffuse peritonitis are rare.

We monitored the changes in TAT, PIC, t-PAIC, and sTM in four cases of acute diffuse peritonitis, and the results showed that they had the same characteristics of early elevated TAT, which suggested the onset of a hypercoagulable state ( Fig. 2 ), resulting in anticoagulant therapy administration. Conventional coagulation function markers, including PT, fibrinogen, D-dimer, and PLT, did not show obvious changes in the early stages of the disease ( Fig. 1A, B ). This finding is consistent with the previous cases, including the infants with sepsis. 5 9 Interestingly, TAT levels were normal in our patients with anal atresia or post-NEC stricture after surgery without signs of peritonitis, suggesting that surgical trauma could not induce TAT elevation. Therefore, TAT elevation may be a potential biomarker for predicting hypercoagulability with high sensitivity and specificity in the postoperative period.

In the early stage of NEC, coagulation activation typically precedes fibrinolytic activation, resulting in elevated TAT with normal PIC levels (<0.8 μg/mL). 10 11 This pattern was observed in cases 2 to 4, reflecting localized intestinal inflammation without systemic fibrinolytic activation. In contrast, case 1 exhibited a sequential activation pattern of coagulation and fibrinolytic systems. The infant suffered severe perinatal asphyxia and subsequently developed NEC IIIA on day 3, accompanied by elevations in both TAT and PIC. Endothelial injury induced by ischemia-reperfusion immediately after birth likely triggered the release of tissue factor and tissue-type plasminogen activator, resulting in transient activation of both systems. 12 13 The subsequent onset of NEC further aggravated endothelial dysfunction, promoting upregulation of procoagulant mediators and secondary tissue-type plasminogen activator release. 14 15 Infants without antecedent asphyxia, however, rarely exhibit a comparable rise in PIC, as endothelial integrity and hepatic clearance of fibrinolytic products remain largely preserved. 16 Collectively, these findings suggest that severe perinatal asphyxia serves as a priming event for the coagulation and fibrinolytic systems, whereas NEC serves as a second insult that amplifies both thrombin generation and fibrinolytic activation.

In cases 1 to 3, sTM increased after treatment even though the re-examination at a time when CRP had already normalized, indicating that endothelial injury and ongoing repair continued beyond the resolution of systemic inflammation. In infants, endothelial regeneration and glycocalyx restoration progress slowly, leading to a temporal dissociation between systemic clinical recovery and microvascular repair. 17 18 Moreover, during the ensuing repair phase, endothelial regeneration and neovascular responses may promote transient shedding of sTM from the endothelial surface, leading to a temporary rise in circulating levels. 19 In contrast, case 4 exhibited minimal sTM variation, implying limited endothelial involvement and relatively mild microvascular injury.

In our neonatal intensive care unit, these biomarkers have been routinely used for serial monitoring of coagulation function in critically ill patients. 20 21 Low-molecular-weight heparin (LMWH) was added once TAT levels increased. The dose of LMWH was adjusted based on the level of anti-Xa to maintain the level within a range of 0.1 to 0.5 U/mL. Anticoagulant therapy with LMWH was discontinued if the TAT level returned to normal. A recent retrospective study demonstrated that coagulation disorders are associated with the severity of NEC. 22 Therefore, we speculated that early anticoagulation therapy could delay disease progression. The duration of gastrointestinal rest in case 2 was only 4 days after surgery, which was significantly shorter than the recommended 7 to 10 days. 23 24 Early initiation of enteral feeding (<5 days) is safe, may shorten hospital stay, and reduce the treatment costs of neonatal NEC. 25 In case 3, enteral feeding was initiated 6 days after surgery, similar to that reported in another study. The time taken to reach full enteral feeding was 12 days, which was much shorter than the previously reported 20 to 38 days. 26 The hospitalization duration was 23 days, whereas the previously reported hospitalization duration for neonatal meconium peritonitis was approximately 48 days on average in Canada, 27 57 to 84 days in Japan, and 71 to 73.5 days in Hong Kong. 28 The reported average hospitalization duration for congenital defects of the gastric musculature in preterm neonates with gestational age < 34 weeks was approximately 30 days (21–36.5 days), which was the same as in case 4. However, the time to reach full enteral feeding was 11 days, which was much shorter than the reported 18 to 34 days. 29 We speculate that the intestinal microcirculation was significantly improved by timely surgery, early anticoagulation, and precision antibiotics, thus establishing a foundation for early enteral feeding. Altogether, our anticoagulant therapy based on the levels of TAT, PIC, t-PAIC, and sTM in four infants with acute diffuse peritonitis improved disease outcomes. However, clinical trials with sufficient sample sizes are needed to evaluate the diagnostic, therapeutic, and prognostic predictive values of TAT, PIC, t-PAIC, and sTM in neonatal infectious diseases. Clinical research, such as the present report, is required to study the characteristics of DIC secondary to acute diffuse peritonitis with different etiologies and pathogens.

## Conclusion

Due to their immature coagulation systems, neonates have a higher risk of coagulation dysfunction caused by various diseases. In neonatal infectious diseases such as acute infectious peritonitis, early recognition and treatment of the abnormal changes in the coagulation and fibrinolytic systems are important to improve the intestinal microcirculation and the prognosis. The coagulation markers TAT, PIC, t-PAIC, and sTM appear to predict the early phase of DIC and guide anticoagulant therapy effectively.

## References

[1] Mehl S C Portuondo J I Tian Y Hospital variation in mortality and failure to rescue after surgery for high-risk neonatal diagnoses Neonatology 2024 121 01 34 45 37844560 10.1159/000533825

[2] Bell M J Peritonitis in the newborn–current concepts Pediatr Clin North Am 1985 32 05 1181 1201 3897986 10.1016/s0031-3955(16)34900-8

[3] de la Hunt M N The acute abdomen in the newborn Semin Fetal Neonatal Med 2006 11 03 191 197 16616711 10.1016/j.siny.2006.01.004

[4] Go H Ogasawara K Maeda H Predicting neonatal mortality with a disseminated intravascular coagulation scoring system Int J Hematol 2023 117 02 278 282 36367668 10.1007/s12185-022-03476-9

[5] Li J Zhou J Ren H Clinical efficacy of soluble thrombomodulin, tissue plasminogen activator inhibitor complex, thrombin-antithrombin complex,α2-plasmininhibitor-plasmin complex in pediatric sepsisClin Appl Thromb Hemost 2022;28:10760296221102929 10.1177/10760296221102929 PMC9134456 35603624

[6] Mei H Jiang Y Luo L Evaluation the combined diagnostic value of TAT, PIC, tPAIC, and sTM in disseminated intravascular coagulation: a multi-center prospective observational study Thromb Res 2019 173 20 26 30458338 10.1016/j.thromres.2018.11.010

[7] Wang W Gao Y Qiao Y Anticoagulant therapy in neonatal acute infectious peritonitis based on the TAT, PIC, tPAIC, and sTM: a new case series Research Square 2024 10.21203/rs.3.rs-4712743/v1 PMC12757086 41488575

[8] Xiang L Ren H Wang Y Clinical value of pediatric sepsis-induced coagulopathy score in diagnosis of sepsis-induced coagulopathy and prognosis in children J Thromb Haemost 2021 19 12 2930 2937 34407568 10.1111/jth.15500

[9] Mautone A Giordano P Montagna O Quercia M Altomare M De Mattia D Coagulation and fibrinolytic systems in the ill preterm newborn Acta Paediatr 1997 86 10 1100 1104 9350893 10.1111/j.1651-2227.1997.tb14816.x

[10] Sokou R Mantzios P Tsantes A G Assessment of hemostatic profile in neonates with necrotizing enterocolitis using rotational thromboelastometry (ROTEM) Pediatr Res 2024 95 06 1596 1602 38092966 10.1038/s41390-023-02958-8

[11] Namachivayam K MohanKumar K Shores D R Targeted inhibition of thrombin attenuates murine neonatal necrotizing enterocolitis Proc Natl Acad Sci U S A 2020 117 20 10958 10969 32366656 10.1073/pnas.1912357117 PMC7245134

[12] Tsaousi M Sokou R Pouliakis A Hemostatic status of neonates with perinatal hypoxia, studied via NATEM in cord blood samples Children (Basel) 2024 11 07 799 39062248 10.3390/children11070799 PMC11276384

[13] Sokou R Tsantes A G Konstantinidi A Rotational thromboelastometry in neonates admitted to a neonatal intensive care unit: a large cross-sectional study Semin Thromb Hemost 2021 47 07 875 884 34130345 10.1055/s-0041-1729964

[14] Madoiwa S Recent advances in disseminated intravascular coagulation: endothelial cells and fibrinolysis in sepsis-induced DIC J Intensive Care 2015 3 8 27408725 10.1186/s40560-015-0075-6 PMC4940964

[15] Giuliani S Tan Y W Zheng D Coagulation gene expression profiling in infants with necrotizing enterocolitis J Pediatr Gastroenterol Nutr 2016 63 06 e169 e175 27050058 10.1097/MPG.0000000000001215

[16] Scientific and Standardization Committee on DIC, and the Scientific and Standardization Committee on Perioperative and Critical Care of the International Society on Thrombosis and Haemostasis Iba T Levy J H Warkentin T E Thachil J van der Poll T Levi M Diagnosis and management of sepsis-induced coagulopathy and disseminated intravascular coagulation J Thromb Haemost 2019 17 11 1989 1994 31410983 10.1111/jth.14578

[17] Iba T Maier C L Helms J Ferrer R Thachil J Levy J H Managing sepsis and septic shock in an endothelial glycocalyx-friendly way: from the viewpoint of surviving sepsis campaign guidelines Ann Intensive Care 2024 14 01 64 38658435 10.1186/s13613-024-01301-6 PMC11043313

[18] Lin J J Hsiao H J Chan O W Wang Y Hsia S H Chiu C H Increased serum thrombomodulin level is associated with disease severity and mortality in pediatric sepsis PLoS One 2017 12 08 e0182324 28771554 10.1371/journal.pone.0182324 PMC5542536

[19] Okamoto T Hattori M Katsube Y Hornerin expressed on endothelial cells via interacting with thrombomodulin modulates vascular inflammation and angiogenesis Biochim Biophys Acta Mol Cell Res 2025 1872 02 119891 39689828 10.1016/j.bbamcr.2024.119891

[20] Zhang W Liu F Liang E Zhang L Evolution of treatment modalities for disseminated HAdV infection in neonates Pediatrics 2024 154 04 e2024066677 39238444 10.1542/peds.2024-066677

[21] Wang W Jiang X Wu W Zhang L Case report: primary segmental volvulus in an infant Front Pediatr 2025 13 1.707716E6 10.3389/fped.2025.1707716 PMC12657436 41321463

[22] Kang C Zhang R Wang G Simple scoring system that predicts the need for surgical intervention in infants with necrotizing enterocolitis Arch Med Res 2023 54 01 37 44 36400576 10.1016/j.arcmed.2022.11.002

[23] Evidence-Based Medicine Group [Clinical guidelines for the diagnosis and treatment of neonatal necrotizing enterocolitis (2020)] Zhongguo Dang Dai Er Ke Za Zhi 2021 23 01 1 11 33476530 10.7499/j.issn.1008-8830.2011145 PMC7818154

[24] Neu J Walker W A Necrotizing enterocolitis N Engl J Med 2011 364 03 255 264 21247316 10.1056/NEJMra1005408 PMC3628622

[25] Hock A M Chen Y Miyake H Koike Y Seo S Pierro A Initiation of enteral feeding after necrotizing enterocolitis Eur J Pediatr Surg 2018 28 01 44 50 28837997 10.1055/s-0037-1604436

[26] Nakagawa Y Uchida H Amano H Safety and feasibility of primary radical surgery for meconium peritonitis considering patients' general condition and perioperative findings Nagoya J Med Sci 2022 84 01 148 154 35392019 10.18999/nagjms.84.1.148 PMC8971046

[27] Shinar S Agrawal S Ryu M Fetal meconium peritonitis - prenatal findings and postnatal outcome: a case series, systematic review, and meta-analysis Ultraschall Med 2022 43 02 194 203 32575129 10.1055/a-1194-4363

[28] Wong C WY Wong K KY Meconium peritonitis: a 22-year review in a tertiary referral center J Pediatr Surg 2022 57 08 1504 1508 34794810 10.1016/j.jpedsurg.2021.10.006

[29] Feng Y Zhou C Li B Clinical diagnosis and treatment of congenital defects of gastric musculature: a report of 104 cases J Clin Ped Surg. 2021 20 11 5

